# The revictimization of older Mexican women: understanding the accumulation of multiple victimizations throughout a lifetime

**DOI:** 10.1186/s12877-021-02734-5

**Published:** 2022-01-10

**Authors:** Liliana Giraldo-Rodríguez, Dolores Mino-León, Sergio Olinsser Aragón-Grijalva, Marcela Agudelo-Botero

**Affiliations:** 1 Dirección de Investigación, Instituto Nacional de Geriatría, Mexico City, Mexico; 2 Unidad de Investigación en Epidemiología Clínica, Hospital de Especialidades, Centro Médico Nacional “Siglo XXI”, Instituto Mexicano del Seguro Social, Mexico City, Mexico; 3 Maestría en Ciencias Matemáticas. Instituto de Investigaciones en Matemáticas Aplicadas y en Sistemas. Universidad Nacional Autónoma de Mexico, Mexico City, Mexico; 4 Centro de Investigación en Políticas, Población y Salud, Facultad de Medicina, Universidad Nacional Autónoma de México, Mexico City, Mexico

**Keywords:** Domestic violence, Child abuse, Intimate partner violence, Elder abuse, Multiple victimization, Revictimization, Mexico

## Abstract

**Background:**

The victimization of women constitutes a human rights violation and a health risk factor. The central objectives of this study were to analyze the probability of revictimization among older adult Mexican women and to examine whether child abuse (CA) and/or intimate partner violence (IPV) are associated with a greater risk of elder abuse (EA) victimization.

**Methods:**

We conducted a secondary data analysis of 18416 women 60 and older, based on data from the National Survey on the Dynamics of Household Relationships (2016), which is national and subnational representative. A descriptive analysis was carried out using retrospective self-reports of victimization experiences (CA, IPV, and EA). The prevalence of victimization and multiple victimizations in the various stages of the lives of women, as well as of revictimization among older adult women were obtained. Bayesian logistic regression models were used to examine the associations between victimization, multiple victimization, and EA victimization.

**Results:**

A total of 17.3% of the older adult women reported EA in the last year; of these, 81.0% had been revictimized and 14.0% reported CA, IPV, and EA. The risk of EA rose among women who reported a combination of psychological and sexual CA, and psychological, physical and sexual CA and psychological and sexual IPV, and a psychological, economic, physical and sexual IPV. EA was higher among women who had suffered more than one type of violence.

**Conclusion:**

CA and IPV, particularly sexual abuse and psychological violence, can be risk factors for EA. Screening tools used to prevent and detect EA should include questions about domestic violence over the course of a person’s lifetime.

## Background

The victimization of women constitutes a clear human rights violation and a physical and mental health risk factor [1]. The most commonly reported violence perpetrated against women is family and domestic violence—that is, violence largely between family members and intimate partners, usually, though not exclusively, taking place in the home. Family and domestic violence include child abuse (CA), intimate partner violence (IPV), and elder abuse (EA) [1, 2]. The research on family and domestic violence shows how women who have been abused once are more likely to be victimized again [3, 4].

Violence against older women is a growing problem in societies. Mexico is through a prolonged time of insecurity, which has increased women’s vulnerability to all forms of violence, both inside and outside the family space. According to the National Institute of Statistics and Geography in Mexico (In Spanish, *Instituto Nacional de Estadística y Geografía* [INEGI]), 66.1% of women 15 and older have experienced at least one incident of violence throughout their lives, and 39.8% have experienced violence during the last year [5]. Between 12 and 20% of older Mexican women (60 and older) have reported having suffered recent (past 12 months) psychological, physical, financial, or economic violence; neglect, or sexual abuse [6, 7]. Furthermore, older women face social, economic, and health disadvantages. This means greater poverty, a high prevalence of chronic diseases, geriatric syndromes, frailty, disability, and a lower quality of life—factors that have been associated with elder abuse [8–10]. Additionally, as is the case around the world, women in Mexico live longer than men, albeit in poorer health [11].

Mexico passed the General Law on Women’s Access to a Violence-Free Life, bringing the phenomenon of violence against women into the public eye [12]. However, it has proved ineffective and has largely remained a written record of intentions [13]. In the country, there is no system for enforcing justice that has been successful in prosecuting various forms of violence, and many victims refrain from reporting assault out of fear or a lack of knowledge of procedures [14].

Although it is an international priority, no studies in Mexico have analyzed the revictimization of older adult women while considering violence in childhood and in partner relationships. This information may aid in the development of public policy and interventions aimed at preventing, identifying, and managing EA. The central objectives of this study were to analyze the probability of the revictimization of older Mexican adult women and to examine whether child abuse (CA) and/or intimate partner violence (IPV) are associated with a greater risk of elder abuse (EA) victimization. The proposed hypotheses were:

- Hypothesis 1: Women who experienced multiple victimization in childhood were at higher risk for EA.
- Hypothesis 2: Women who experienced multiple victimization in intimate partner relationships were at higher risk for EA.

### The concept of revictimization and previous studies

Violence towards women has been studied from various theoretical and analytical perspectives. Considering the exposure to violence that women experience throughout their lives, there has been a growing interest in recent years in understanding violence against women and seeking data about its nature, dynamics, and characteristics.

According to Scott-Storey (2011), no single type of abuse occurs in isolation from others for women; having a single, isolated abusive experience is frequently the exception rather than the rule. To explain the stories of abuse against women, this author introduces the concept of “cumulative abuse,” which is one of the most frequently used categories of revictimization in the literature [15].

Revictimization refers to the exposure to one or multiple types of violence (psychological, physical, sexual abuse, and negligence) that women are exposed to throughout their lives. This violence may come from one or various perpetrators, in one or several contexts (for example, in the home, in the school, or at work). The term revictimization has served to study the cumulative harmful effects of violence on women’s physical and mental health, as well as to estimate the risk of victimization at a given moment by considering violent events in previous life stages [3, 16–18].

Although revictimization has been studied since the mid-1980s, there is still no agreement on its precise definition, resulting in new proposals. In this context, Bocker and collaborators (2014) proposed the term revictimization to refer to situations in which at least two distinct traumatic events (multiple victimization) occur during two distinct stages of life and are committed by distinct perpetrators (parents, husband, children, and other members of the household) [18].

Most studies of revictimization have consistently shown that survivors of childhood sexual abuse are at a higher risk of victimization later in life than the general population [19]. In this vein, some research suggests that two of three individuals who are sexually victimized will be revictimized [20]. It has also been reported that experiencing multiple types of violence during childhood (including sexual abuse) and experiencing domestic violence are associated with victimization later in life (either IPV or EA) [3, 21–23].

In general, studies of revictimization have focused on the dichotomous CA-IPV analysis. However, few studies have shined light on the phenomenon through older adult. It has been found that exposure to violence in childhood predicts victimization in older adulthood [24], and that experiences of IPV are perpetuated throughout the lives of women, including into old age [25]. IPV may continue into old age or may decline with age, during which point women may begin to experience violence at the hands of another family member [4]. Individuals who had previously experienced psychological CA were four times as likely to experience IPV and EA psychological violence again; individuals who had previously experienced IPV psychological violence were eight times as likely to experience EA psychological violence. The combination of physical and sexual violence was also found to persist across the life course [26].

## Methods

### Data sources

The data for this study is drawn from the 2016 National Survey on the Dynamics of Household Relationships (In Spanish, *Encuesta Nacional sobre la Dinámica de las Relaciones en los Hogares* [ENDIREH-2016]), a national and subnational representative survey that collected information on the physical, economic, sexual, psychological, and patrimonial violence experienced by women 15 and older in their various life environments (family, partner relationships, school, work, and community). It also compiled information about the aggressors and the places where aggressions occurred. This information allows the violence to be measured and characterized, both that which occurs throughout women’s lifetimes and that which has occurred recently (in the last 12 months). Additionally, the ENDIREH-2016 includes a section specifically designed to investigate incidents of sexual, physical, and psychological abuse occurring during childhood, and another on violence directed towards women 60 and older [5].

The ENDIREH has had four rounds. The first was in 2003, conducted by the National Women’s Institute (In Spanish, *Instituto Nacional de Mujeres,* [Inmujeres]), the United Nations Development Fund for Women, and the INEGI. The second survey was conducted in 2006 and provided the information necessary to drive the proposal and approval of the General Law for Women’s Access to a Life Free of Violence. The third survey was conducted in 2011, with the same objectives but new characteristics. The interviewers received training in the needed conceptual, technical, and methodological tools to conduct high quality interviews, while respecting the privacy of the interviews and data. Interviewers also strengthened their skills in assertive communication, empathy, and other abilities needed to react in the face of unexpected and complicated situations, without putting themselves or the informant at risk. Interviews were conducted in a private place, in or outside of the home, and all participants gave their informed consent [5]. ENDIREH data files and documentation are of public use and available at https://www.inegi.org.mx/programas/endireh/2016/.

### Study design and population

**Fig. 1:**
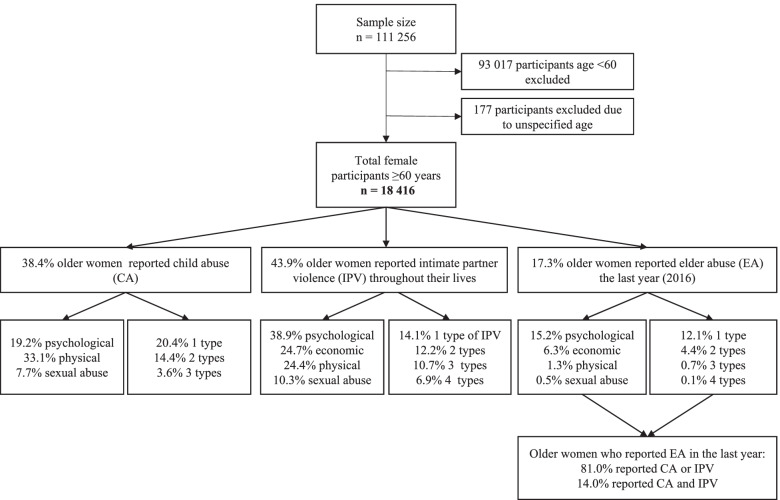
Flowchart of the study population selection. Prevalence of childhood violence, intimate partner violence and elder abuse of older Mexican women

This is a cross-sectional study based on a subsample of women 60 and older from the ENDIREH-2016. The sample consisted of women who were married, divorced, separated, living in unmarried partnerships, or widowed at the time of the survey, and they were questioned retrospectively about their experiences with violence throughout their lives (childhood, partner relationships, and old age), as well as in the year preceding the survey (*n* = 18416) (Fig. 1).

### Measures

#### Victimization

CA is abuse that occurs before the age of 15 and includes psychological, physical, and sexual abuse. IPV is violence at the hands of one’s current or previous partner/s and was divided into psychological, economic, physical, and sexual abuse. EA referred to violence experienced at 60 and older, and may include psychological, economic, physical, and sexual abuse (during the last twelve months) at the hands of close family members or people with whom the older woman lives aside from their current partner. It should be noted that economic violence involved actions or omissions that affect the economic survival of women, as well as acts of coercion and dispossession of goods, material resources or property.

#### Multiple victimization

This occurs when there are two or more types of violence in some stage of life (childhood, partner relationships, or old age).

#### Revictimization

This refers to when an older adult woman experienced at least one type of violence in two or more stages of her life: CA-EA, IPV-EA, or CA-IPA-EA.

### Variables

#### Dependent variable

EA was considered to have occurred when there was at least one affirmative response to a question regarding four types of violence (psychological, economic, physical, and sexual abuse) during the last 12 months by a relative or acquaintance. The responses were dichotomous: “0-no violence” and “1-violence”.

### Covariables

#### Antecedents of victimization

CA included the types psychological, physical, and sexual abuse, as well as their combinations (psychological/physical, psychological/sexual, physical/sexual, and psychological/physical/sexual abuse). IPV included psychological, economic, physical, and sexual abuse, and their combinations (psychological/economic, psychological/physical, psychological/sexual, economic/physical, economic/sexual, physical/sexual, psychological/economic/physical, psychological/economic/sexual, psychological/physical/sexual, economic/physical/sexual, and psychological/economic/physical/sexual violence).

#### Sociodemographic and health variables

Age (60–69, 70–79, 80+ years); ethnicity (speaking an indigenous language); educational level (no schooling, elementary school, high school and undergraduate); marital status (unmarried/single, married/living-in, separated/divorced); economic dependence (economically independent and without economic dependents; economically independent with economic dependents; economically dependent without economic dependents, and economically dependent with economic dependents); and disability and/or disease (having reported having a disease and/or disability that prevents mobility without assistance).

### Statistical analysis

First, a descriptive analysis was carried to obtain the prevalence of victimization and multiple victimization in the various stages of older adult women’s lives, as well as the prevalence of revictimization in old age. Frequencies and percentages were used for the categorical variables.

Second, a Bayesian analysis was used to estimate the conditional probabilities of revictimization of older adult women given an antecedent of CA and/or IPV. The Bayes theorem was used: *p*(A|B) = (*p*(A∩B))/(*p*(B)), where *p*(A|B) is the posterior probability; that is, the probability of *A* given *B.* For this study, *p* (EA|IPV, CA) = (*p* (EA∩IPV∩CA))/(*p* (IPV∩CA)), where *p* (EA|IPV, CA) is the conditioned probability of experiencing EA given a history of IPV and/or CA. Monte Carlo simulation was calculated, and the credible interval (CI) for each conditional probability [27].

Third, Bayesian logistic regression models were calculated (without and with adjustment variables) (age, ethnicity, education level, economic dependence, and disease and/or disability), to estimate the association of multiple victimization in childhood and intimate relationships with EA. The likelihood distribution function was modeled as logistic, and the priors were modeled as normal ~ (0,10,000). Markov chain Monte Carlo (MCMC) interactions were set in 12500. The results are reported in terms of odds ratio (OR) and 95% credibility interval. For the analysis, the statistical package Stata version 16.1 (Stata Corp, 2019) and R Programming Language version 4.0.3 (*R Core Team*, 2020) were used.

## Results

### Sociodemographic and health characteristics of the sample population

Table 1 summarizes the characteristics of the sample. Most of the participants were 60–69 years, 27.5% spoke an indigenous language, 50.5% had elementary school education, 25.5% had no schooling, and 51.0% had a partner. A total of 50.3% were economically dependent on another person(s) and had no economic dependents, 19.0% were economically dependent on others and other people were economically dependent on them, and a third were economically independent. Most of the women did not report having any disease and/or disability that limited their mobility (83.2%).

**Table 1:** Elder abuse in past 12 months. Sociodemographic and health characteristics of older Mexican women

	Elder abuse (past 12 months)		
	No	Yes	Total
	*n* = 15332	*n* = 3084	*n* = 18416
**Age** (years)			
60–69	83.8	16.2	57.3
70–79	81.2	18.8	30.0
80+	81.7	18.3	12.7
**Ethnicity** (speaking an indigenous language)			
No	83.4	16.6	72.5
Yes	81.0	19.0	27.5
**Educational level**			
No schooling	79.7	20.3	25.5
Elementary school	82.2	17.9	50.5
High school and undergraduate	87.2	12.8	24.1
**Marital status**			
Married/living-in	85.0	15.0	51.0
Separated/divorced	80.4	19.6	44.0
Unmarried/single	82.6	17.4	5.1
**Economic dependence**			
Economically independent and without economic dependents	80.5	19.5	17.3
Economically independent with economic dependents	77.6	22.4	13.4
Economically dependent without economic dependents	85.6	14.4	50.3
Economically dependent with economic dependents	80.8	19.2	19.0
**Disease and/or disability**			
No	84.1	15.9	83.2
Yes	76.2	23.8	16.8
**Child abuse** (CA)			
No	88.4	11.6	61.6
Psychological	74.5	25.5	2.5
Physical	82.5	17.5	15.5
Sexual	66.4	33.7	2.4
Psychological/physical	68.5	31.5	12.7
Psychological/sexual	67.2	32.8	0.4
Physical/sexual	65.2	34.8	1.3
Psychological/physical/sexual	61.1	38.9	3.6
**Intimate partner violence** (IPV)			
No	89.8	10.2	56.1
Psychological	85.6	14.4	9.6
Economic	85.7	14.3	2.6
Physical	82.1	17.9	1.8
Sexual	82.2	17.8	0.2
Psychological/economic	75.9	24.2	5.5
Psychological/physical	75.1	25.0	5.8
Psychological/sexual	57.9	42.1	0.6
Economic/physical	74.5	25.5	0.4
Economic/sexual	79.2	20.8	0.1
Physical/sexual	83.3	16.7	0.0
Psychological/economic/physical	65.7	34.3	8.1
Psychological/economic/sexual	67.5	32.5	1.1
Psychological/physical/sexual	63.0	37.0	1.4
Economic/physical/sexual	100.0	0.0	0.0
Psychological/economic/physical/sexual	61.3	38.7	6.9
**Multiple victimization CA**			
1 type	79.6	20.4	20.4
2 types	68.2	31.8	14.4
3 types	61.1	38.9	3.6
**Multiple victimization IPV**			
1 type	85.1	14.9	14.1
2 types	74.7	25.3	12.3
3 types	65.6	34.4	10.7
4 types	61.3	38.7	6.9

### Prevalence of victimization, multiple victimization, and revictimization

A total of 38.4% of the older women reported CA (19.2% psychological, 33.1% physical and 7.7% sexual abuse); 43.9% reported IPV (38.9% psychological, 24.7% economic, 24.4% physical and 10.3% sexual abuse); and 17.3% of the older women reported EA by family members or acquaintances in the last year (15.2% psychological, 6.3% economic, 1.3% physical and 0.5% sexual abuse) (Fig. 1).

Of the women who reported experiencing CA, 15.5% reported physical CA, 12.7% psychological/physical CA, 3.6% psychological/physical/sexual CA, 2.5% psychological CA, 2.4% sexual CA and, 1.3% physical/sexual CA; 14.4% of the women had experienced at least two types of CA and 3.6% three types of CA. Of the women who reported experiencing IPV throughout their lives, 9.6% reported psychological violence, 8.1% psychological/economic/physical, 6.9% psychological/economic/physical/sexual, 5.8% psychological/physical, 5.5% psychological/economic, 2.6% economic, 1.8% physical, 1.4% psychological/physical/sexual, 1.1% psychological/economic/sexual, 0.6% psychological/sexual and, 0.4% economic/physical violence. Overall, 12.3% of the women had experienced at least two types of IVP, 10.7% three types of IVP and, 6.9% four types of IVP (Table 1). Of the women who reported having suffered EA in the last 12 months, 10.1% reported psychological abuse, 3.8% psychological/economic, 1.8% economic, 0.5% psychological/economic/physical, 0.5% psychological/physical, 0.1% sexual, 0.1% psychological/sexual, 0.1% psychological/economic/sexual, 0.1% psychological/physical/sexual, 0.1% psychological/economic/physical/sexual and, 0.1% physical abuse. Around of 4.5% of the women had experienced at least two types of EA, 0.7% three types of EA and 0.1% four types of EA (data not shown). Finally, of the older women who reported EA during the last 12 months, 81.0% reported revictimization (CA or IPV) and 14.0% were revictimized in all three stages of life; that is, they suffered CA, IPV, and EA (Fig. 1).

### Probability of revictimization in old age

Before creating Bayesian regression models, we calculated the conditional probabilities of revictimization of older adult women given an antecedent of CA and IPV. The results of the Bayesian analysis between CA, IPV, and EA are summarized in Table 2. The following groups were more likely to be revictimized in old age: older women who reported psychological CA and psychological/physical/sexual IPV; psychological CA and psychological/economic/physical IPV; psychological/sexual CA and psychological/physical IPV; psychological/sexual CA and psychological/economic IPV; and psychological/sexual CA and psychological/economic/physical IPV. Regarding multiple victimization, older women who reported three types of CA and four types of IPV were more likely to experience EA. Meanwhile, the lowest probability of EA occurred in women who reported not having suffered CA or IPV throughout their lives (Table 3).

**Table 2:** Conditional probability of experiencing elder abuse by type in childhood and partner relationships

		Child Abuse							
		Psychological	Physical	Sexual	Psychological/physical	Psychological/sexual	Physical/sexual	Psychological/Physical/sexual	No abuse
**Intimate partner violence**	Psychological	0.24 (0.23–0.26)	0.16 (0.15–0.18)	0.37 (0.34–0.39)	0.20 (0.18–0.22)	d	0.30 (0.28–0.32)	0.38 (0.35–0.40)	0.14 (0.12–0.15)
	Economic	0.15 (0.14–0.17)	0.14 (0.13–0.16)	0.25 (0.23–0.27)	0.30 (0.28–0.32)	c	0.43 (0.41–0.45)	0.50 (0.48–0.52)	0.14 (0.12–0.15)
	Physical	0.18 (0.16–0.20)	0.11 (0.10–0.13)	0.43 (0.41–0.45)	0.26 (0.24–0.28)	a	0.20 (0.18–0.22)	0.27 (0.25–0.29)	0.11 (0.10–0.13)
	Sexual	d	0.11 (0.10–0.13)	a	0.50 (0.48–0.52)	c	c	0.50 (0.48–0.52)	0.23 (0.21–0.25)
	Psychological/economic	0.38 (0.35–0.40)	0.19 (0.18–0.21)	0.45 (0.43–0.48)	0.30 (0.28–0.32)	0.60 (0.58–0.62)	0.38 (0.35–0.40)	0.43 (0.41–0.46)	0.23 (0.21–0.25)
	Psychological/physical	0.19 (0.17–0.21)	0.18 (0.17–0.20)	0.32 (0.30–0.35)	0.28 (0.26–0.30)	0.67 (0.64–0.69)	0.37 (0.35–0.39)	0.42 (0.39–0.44)	0.17 (0.15–0.19)
	Psychological/sexual	0.50 (0.48–0.52)	0.24 (0.22–0.26)	0.40 (0.38–0.42)	0.54 (0.52–0.56)	a	0.20 (0.18–0.22)	0.29 (0.26–0.31)	0.32 (0.30–0.34)
	Economic/physical	0.33 (0.31–0.35)	0.29 (0.26–0.31)	a	0.23 (0.21–0.25)	c	c	0.50 (0.48–0.52)	0.16 (0.14–0.17)
	Economic/sexual	b	d	d	0.50 (0.48–0.52)	c	c	c	0.17 (0.15–0.18)
	Physical/sexual	c	0.20 (0.18–0.22)	d	c	c	d	c	0.33 (0.31–0.36)
	Psychological/economic/physical	0.60 (0.58–0.62)	0.24 (0.22–0.26)	0.40 (0.38–0.43)	0.41 (0.39–0.43)	0.60 (0.58–0.62)	0.35 (0.33–0.37)	0.45 (0.42–0.47)	0.24 (0.22–0.26)
	Psychological/economic/sexual	0.38 (0.35–0.40)	0.38 (0.35–0.40)	0.36 (0.34–0.39)	0.53 (0.51–0.56)	b	0.14 (0.13–0.16)	0.33 (0.31–0.35)	0.21 (0.19–0.23)
	Psychological/physical/sexual	0.67 (0.64–0.69)	0.41 (0.39–0.44)	0.27 (0.25–0.29)	0.40 (0.38–0.43)	d	0.50 (0.48–0.52)	0.46 (0.44–0.49)	0.29 (0.27–0.31)
	Economic/physical/sexual	c	d	c	d	c	c	c	d
	Psychological/economic/physical/sexual	0.58 (0.55–0.60)	0.33 (0.3–0.35)	0.28 (0.26–0.3)	0.46 (0.43–0.48)	0.38 (0.36–0.41)	0.40 (0.38–0.42)	0.57 (0.55–0.59)	0.28 (0.26–0.30)
	No violence	0.15 (0.13–0.16)	0.12 (0.10–0.13)	0.20 (0.18–0.22)	0.20 (0.18–0.22)	0.50 (0.48–0.52)	0.27 (0.25–0.29)	0.39 (0.37–0.41)	0.08 (0.07–0.10)

Ten thousand simulations of 1842 samples were performed for each of the probabilities obtained by the Bayesian analysis. Two-tailed 95% credibility interval with Monte Carlo simulation generated random variables with uniform distribution similar to the results observed by Bayesian analysis

The figures were truncated to two digits

^a^Single event with a probability of violence of 1: CA, by an IPV and in EA (imprecise probability of EA given the number of reported cases; not necessarily 1)

^b^Non-single event with a probability of violence of 1: CA, by an IPV and in old age (high probability of EA, but given the number of cases reported, the probability is not necessarily equal to 1)

^c^No occurred CA, IPV or EA; probability of violence 0 (imprecise probability of EA given the absence of reported cases; the probability is not necessarily equal to 0)

^d^No occurred EA, but CA and IPV did (low probability of EA, but given the number of cases reported, the probability is not necessarily equal to 0)

**Table 3:** Conditional probability of experiencing elder abuse by multiple victimization in childhood and partner relationships

		Child Abuse			
		1 type	2 types	3 types	No violence
**Intimate partner violence**	1 type	0.18 (0.16–0.19)	0.24 (0.22–0.26)	0.38 (0.36–0.40)	0.14 (0.12–0.15)
	2 types	0.23 (0.21–0.25)	0.31 (0.29–0.33)	0.42 (0.39–0.44)	0.20 (0.18–0.22)
	3 types	0.31 (0.29–0.34)	0.41 (0.39–0.44)	0.44 (0.42–0.46)	0.24 (0.22–0.26)
	4 types	0.34 (0.32–0.36)	0.45 (0.42–0.47)	0.57 (0.55–0.59)	0.28 (0.26–0.30)
	No violence	0.13 (0.11–0.14)	0.21 (0.19–0.23)	0.39 (0.37–0.41)	0.08 (0.07–0.10)

Ten thousand simulations of 1842 samples were performed for each of the probabilities obtained by the Bayesian analysis

Two-tailed 95% credibility interval obtained by Monte Carlo simulation generated random variables with distributions similar to the results observed by Bayesian analysis

The figures were truncated to two digits

### Associations between elder abuse and multiple victimization in childhood and/or partner relationships

Bayesian logistic regression models (without and with adjustment), revealed that multiple victimization, both in childhood and in partner relationships, increased the risk of experiencing EA. After adjusting for sociodemographic and health characteristics, who reported the combination of psychological/sexual CA had higher odds of experiencing EA (adjusted odds ratio [aOR], 3.89; 95% CI, 3.18–4.58), followed by the combination of psychological/physical/sexual CA (aOR, 3.55; 95% CI, 3.16–3.99) and the combination of physical/sexual CA (aOR, 2.62; 95% CI, 2.40–2.88). For older women who reported IPV, the combination of psychological/sexual violence had higher odds of EA (aOR, 3.93; 95% CI, 3.09–4.91), followed by psychological/economic/physical/sexual (aOR, 3.51; 95% CI, 3.13–3.91), psychological/economic/sexual (aOR, 3.48; 95% CI, 3.08–3.92) and psychological/physical/sexual combinations (aOR, 3.35; 95% CI, 2.83–3.87) (Table 4). In addition, the risk of experiencing EA increased when women suffered multiple victimization, the highest risk was observed for women who reported having experienced three types of CA and for those who reported having experienced four types of IPV (Table 5).

**Table 4:** Associations between elder abuse and multiple victimization in childhood and/or partner relationships

	Model 1			Model 2		
	OR	SD	95% CI	OR	SD	95% CI
**Age** (ref. 60–69 years)						
70–79				1.18	0.05	1.10–1.29
80+				1.24	0.07	1.10–1.38
**Ethnicity** (ref. Not speaking an indigenous language)						
Yes				1.02	0.04	0.95–1.11
**Educational level** (ref. No schooling)						
Elementary school				0.85	0.04	0.77–0.94
High school and undergraduate				0.59	0.03	0.54–0.66
**Economic dependence** (ref. Economically independent and without economic dependents)						
Economically independent with economic dependents				1.19	0.64	1.07–1.32
Economically dependent without economic dependents				0.65	0.03	0.59–0.71
Economically dependent with economic dependents				0.84	0.39	0.76–0.92
**Disease and/or disability** (ref. no)						
Yes				1.55	0.66	1.42–1.69
**Child abuse** (ref. no)						
Psychological	2.34	0.25	1.93–2.94	2.21	0.14	1.94–2.50
Physical	1.24	0.06	1.11–1.36	1.28	0.06	1.17–1.42
Sexual	2.16	0.27	1.72–2.75	2.32	0.16	2.02–2.64
Psychological-physical	2.30	0.11	2.11–2.52	2.21	0.08	2.06–2.37
Psychological-sexual	3.88	0.36	3.16–4.59	3.89	0.37	3.18–4.58
Physical-sexual	2.10	0.22	1.67–2.55	2.62	0.12	2.40–2.88
Psychological-physical-sexual	3.59	0.25	3.14–4.11	3.55	0.22	3.16–3.99
**Intimate partner violence** (ref. no)						
Psychological	1.52	0.09	1.36–1.71	1.61	0.10	1.44–1.79
Economic	1.57	0.18	1.26–1.96	1.56	0.14	1.34–1.88
Physical	1.27	0.15	1.04–1.64	1.43	0.15	1.19–1.69
Sexual	2.16	0.34	1.55–2.84	2.46	0.12	2.23–2.72
Psychological-economic	2.49	0.19	2.13–2.90	2.51	0.16	2.20–2.86
Psychological-physical	1.98	0.15	1.71–2.27	1.99	0.11	1.80–2.21
Psychological-sexual	3.29	0.37	2.61–3.98	3.93	0.47	3.09–4.91
Economic-physical	2.85	0.25	2.34–3.30	2.33	0.12	2.10–2.58
Economic-sexual	2.94	0.44	2.20–3.83	2.50	0.14	2.23–2.78
Physical-sexual	1.72	0.47	0.82–2.54	1.43	0.13	1.19–1.68
Psychological-economic-physical	3.04	0.20	2.66–3.45	2.94	0.17	2.61–3.28
Psychological-economic-sexual	3.01	0.24	2.61–3.53	3.48	0.21	3.08–3.92
Psychological-physical-sexual	3.50	0.30	2.95–4.08	3.35	0.26	2.83–3.87
Economic-physical-sexual	–			–		
Psychological-economic-physical-sexual	3.74	0.27	3.20–4.27	3.51	0.21	3.13–3.91

*OR* odds ratio, *SD* standard deviation, *CI* credible interval

**Table 5:** Associations between elder abuse and multiple victimization in childhood and/or partner relationships

	Model 3			Model 4		
	OR	SD	95% CI	OR	SD	95% CI
**Age** (ref. 60–69 years)						
70–79				1.20	0.06	1.10–1.32
80+				1.22	0.08	1.09–1.38
**Ethnicity** (ref. Not speaking an indigenous language)						
Yes				1.05	0.05	0.97–1.17
**Educational level** (ref. No schooling)						
Elementary school				0.84	0.03	0.77–0.91
High school and undergraduate				0.60	0.03	0.54–0.66
**Economic dependence** (ref. Economically independent and without economic dependents)						
Economically independent with economic dependents				1.19	0.07	1.06–1.34
Economically dependent without economic dependents				0.66	0.02	0.62–0.71
Economically dependent with economic dependents				0.87	0.04	0.80–0.96
**Disease and/or disability** (ref. no)						
Yes				1.54	0.07	1.41–1.68
**Child abuse** (ref. no)						
1 type	1.46	0.08	1.32–1.61	1.46	0.06	1.35–1.58
2 types	2.33	0.12	2.09–2.58	2.27	0.10	2.10–2.47
3 types	3.66	0.37	3.01–4.45	3.58	0.30	3.04–4.23
**Intimate partner violence** (ref. no)						
1 type	1.56	0.10	1.38–1.75	1.53	0.08	1.38–1.69
2 types	2.28	0.13	2.02–2.57	2.24	0.10	2.05–2.45
3 types	3.16	0.20	2.78–3.56	3.13	0.18	2.81–3.50
4 types	3.78	0.26	3.31–4.29	3.58	0.22	3.19–4.05

*OR* odds ratio, *SD* standard deviation, *CI* credible interval

## Discussion

This is the first study in Mexico to link older women’s experience of violence to their prior antecedents of violence in childhood and in partner relationships. The findings support the hypothesis that exposure to CA and IPV is a risk factor for revictimization in later life; additionally, the types and combinations of violence, as well as multiple victimization, all have a cumulative effect on the likelihood of suffering violence in later life. These findings were consistent with previous studies in which violence in childhood was linked to the risk of revictimization in later stages of life [3, 21, 28, 29]. Another study discovered that women exposed to violence as children were more likely to become victims of domestic violence as adults [30].

This analysis also revealed that the combination of psychological violence and sexual abuse, both in childhood and in intimate relationships, significantly increased the risk of experiencing violence in old age. Other authors have also reported that experiencing psychological violence and sexual abuse in childhood has a strong association with the risk of victimization in old age [24, 31]. The risk of EA revictimization also increased when physical violence occurred in combination with other types of CA and IPV. Similar results were observed in a European study in which it was reported that women who suffered physical violence or sexual abuse in childhood were more likely to be victims of violence in adulthood; however, physical violence experienced before the age of 15 had a lower association than sexual abuse with all types of violence at other times in life [29].

Sexual abuse, both in childhood and in adulthood, was the least prevalent type of violence reported in this study, but it was a strong predictor of violence in old age, especially, when it was combined with other types of violence in childhood and in partner relationship. Aakvaag and collaborators (2017), found that women who had been sexually abused experienced a higher incidence of violence in childhood and tended to experience other types of violence more frequently than women who did not suffer sexual abuse, and this was observed, mainly, when the father was responsible for the sexual abuse. In addition, the strongest association of revictimization with prior violence was found among women who suffered multiple types of violence in childhood [3]. When combined with other types of violence, psychological violence in childhood or in a partner relationship was a predictor of revictimization in old age. It has been found that in childhood, psychological violence has as strong a relationship with revictimization as sexual abuse, and has a long-term effect in that it increases the risk that women will suffer violence throughout their lives at the hands of either their partner or another person [29].

Additionally, multiple childhood victimization experiences and partner relationships were identified as risk factors for EA in a staggered fashion, i.e., they were associated with the presence of violence in old age. Till-Tentschert (2017) reported that repeated experiences of violence in childhood significantly increase the probability that a woman will suffer any type of violence later in life [29]. However, it should be noted that the author defined repetitive violence in terms of frequency (once, more than once) rather than the sum of the types of violence; nonetheless, the findings agree that when women experience a greater number of incidents of violence, the risk of violence in later stages of life and of revictimization increases significantly [29].

Finally, in the field of elder abuse, progress has been made in determining the factors that are associated with victimization in old age. However, little is known about the factors associated with revictimization. For example, some studies have found that deficits in the ability to recognize risks, attachment anxiety, self-efficacy, dissociation, assertiveness and feelings of guilt and shame explain, in part, experiences of violence throughout life [17, 18, 20]. Most studies on elder abuse have found that problems with stress and coping, attitude, experiencing or witnessing violence in other stages of life, depression, and loneliness are risk factors [32, 33], but studies did not distinguish between victimized and revictimized people. Therefore, for future research, it is important to examine these factors more deeply from different theoretical and methodological perspectives.

## Limitations

Due to the complexity of this topic and the fact that this study was based on secondary and cross-sectional data, the results should be interpreted with the following cautions: (1) it is possible that women who have experienced violence do not report it due to fear, embarrassment, or other factors, resulting in underreporting [34]; (2) victims of violence often have recall biases due to traumatic experiences, which leads to underreporting [35]; (3) people who are not currently experiencing violence tend to forget past episodes, while women who are currently experiencing violence may exaggerate the negativity of previous situations (recall bias) [36, 37]; and (4) people who can recall more recent experiences of violence in adulthood have, in general, a greater awareness and perhaps a greater ability to reflect on incidents that occurred in the past, which can lead to an overestimation of violence [29].

## Conclusion

CA and IPV, particularly sexual abuse and psychological violence, can be risk factors for EA. Screening tools used to prevent and detect EA should include questions about domestic violence over the course of a person’s lifetime. In this regard, additional research is needed to determine the effect of women’s characteristics at various stages of life on revictimization. Similarly, public policies addressing violence against women must take a life-course approach in order to alleviate and reverse its negative consequences.

## Data Availability

The general data that support the findings of this study are available from the National Institute of Statistics and Geography: https://www.inegi.org.mx/programas/endireh/2016/. However, the microdata used in this study are available from the corresponding author on reasonable request.

## Abbreviations

aOR: Adjusted odds ratio
CA: Child abuse
CI: Credible interval
EA: Elder abuse
ENDIREH: Encuesta Nacional sobre la Dinámica de las Relaciones en los Hogares
INEGI: Instituto Nacional de Estadística y Geografía
IPV: Intimate partner violence
MCMC: Markov chain Monte Carlo
OR: Odds Ratio
SD: Standard deviation
